# Histone Acetylation Regulator Gcn5 Mediates Drug Resistance and Virulence of Candida glabrata

**DOI:** 10.1128/spectrum.00963-22

**Published:** 2022-06-06

**Authors:** Shuying Yu, Padmaja Paderu, Annie Lee, Sami Eirekat, Kelley Healey, Liang Chen, David S. Perlin, Yanan Zhao

**Affiliations:** a Center for Discovery and Innovation, Hackensack Meridian Health, Nutley, New Jersey, USA; b Department of Clinical Laboratory, Peking Union Medical College Hospital, Chinese Academy of Medical Sciences, Beijing, People’s Republic of China; c Beijing Key Laboratory for Mechanisms Research and Precision Diagnosis of Invasive Fungal Diseases (BZ0447), Beijing, People’s Republic of China; d Department of Biology, William Paterson University, Wayne, New Jersey, USA; e Department of Medical Sciences, Hackensack Meridian School of Medicine, Nutley, New Jersey, USA; f Department of Microbiology and Immunology, Georgetown University School of Medicine, Washington, DC, USA; University of Iowa Hospitals and Clinics

**Keywords:** *Candida glabrata*, *GCN5*, antifungal resistance, virulence, transcriptomics

## Abstract

Candida glabrata is poised to adapt to drug pressure rapidly and acquire antifungal resistance leading to therapeutic failure. Given the limited antifungal armamentarium, there is an unmet need to explore new targets or therapeutic strategies for antifungal treatment. The lysine acetyltransferase Gcn5 has been implicated in the pathogenesis of C. albicans. Yet how Gcn5 functions and impacts antifungal resistance in C. glabrata is unknown. Disrupting *GCN5* rendered C. glabrata cells more sensitive to various stressors, partially reverted resistance in drug-resistant mutants, and attenuated the emergence of resistance compared to wild-type cells. RNA sequencing (RNA-seq) analysis revealed transcriptomic changes involving multiple biological processes and different transcriptional responses to antifungal drugs in *gcn5*Δ cells compared to wild-type cells. *GCN5* deletion also resulted in reduced intracellular survival within THP-1 macrophages. In summary, Gcn5 plays a critical role in modulating the virulence of C. glabrata and regulating its response to antifungal pressure and host defense.

**IMPORTANCE** As an important and successful human pathogen, Candida glabrata is known for its swift adaptation and rapid acquisition of resistance to the most commonly used antifungal agents, resulting in therapeutic failure in clinical settings. Here, we describe that the histone acetyltransferase Gcn5 is a key factor in adapting to antifungal pressure and developing resistance in C. glabrata. The results provide new insights into epigenetic control over the drug response in C. glabrata and may be useful for drug target discovery and the development of new therapeutic strategies to combat fungal infections.

## INTRODUCTION

Invasive candidiasis is an important fungal infection caused by *Candida* species with high morbidity and mortality rates (1, 2). Although Candida albicans remains the predominant pathogen for invasive candidiasis, infections due to non-C. albicans *Candida* species have increased significantly in the past 2 decades (3, 4), among which Candida glabrata is the most frequently isolated species in the SENTRY antifungal surveillance program (5). The global emergence of C. glabrata is worrisome because this yeast species is poised to adapt to drug pressure and acquire antifungal resistance leading to therapeutic failure (1, 6). The combination of this feature and the highly limited antifungal armamentarium currently available in the clinical setting forms a perfect storm plaguing global public health. Thus, there is an urgent need to explore new targets or therapeutic strategies for antifungal development.

Antifungal resistance studies have focused largely on genetic mutations involving the target site and transcription factors regulating drug efflux pumps, aneuploidy, the upregulation of stress response pathways, and biofilm formation (7). Yet an increasing body of evidence has shown that epigenetic pathways may be important factors contributing to drug resistance via existing or novel mechanisms (8–10). Broadly speaking, epigenetic mechanisms can be either RNA based or chromatin based. The latter consists of both chemical and structural modifications that change the accessibility of transcription factors to specific genomic regions, therefore regulates global transcription (9). Among the most studied chromatin modifications, histone acetylation has been suggested to be critical in dictating the mutational landscape of yeast cells, thus contributing to the development of drug resistance in different fungal pathogens (10–12). Histone acetylation is one of the well-characterized posttranslational modifications (PTMs) involving the addition/removal of acetyl groups to/from lysine residues in the amino tails of histones mediated by lysine acetyltransferases (KATs) and lysine deacetylases (KDACs), respectively (13, 14). It has been suggested that close cooperation of KATs/KDACs with dedicated transcriptional regulators forms a dual-layer network of chromatin-mediated transcriptional control in the major fungal pathogen C. albicans (11). Gcn5 is a pleiotropic KAT constituting the catalytic subunit of the SAGA (Spt-Ada-Gcn5-acetyltransferase) complex that is conserved in eukaryotes (15, 16). Previous studies have shown that Gcn5 plays roles in morphogenesis, pathogenesis, virulence, and the stress response in multiple fungal organisms, including C. albicans (17–21). However, other than two recent studies (22, 23) showing that inhibition of Gcn5 attenuated the emergence of azole resistance in C. glabrata *in vitro* and that a C. glabrata Δ*gcn5* mutant was less virulent than the wild-type (WT) strain in a Galleria mellonella infection model, little is known about how Gcn5 functions and becomes involved in antifungal resistance in this major human fungal pathogen. Hence, we employed multiple strategies and systematically investigated the impact of Gcn5 on the virulence and drug response of C. glabrata.

## RESULTS

### Phenotypic and epigenetic profiles and changes in stress responses and drug susceptibility associated with *GCN5* in C. glabrata.

**FIG 1:**
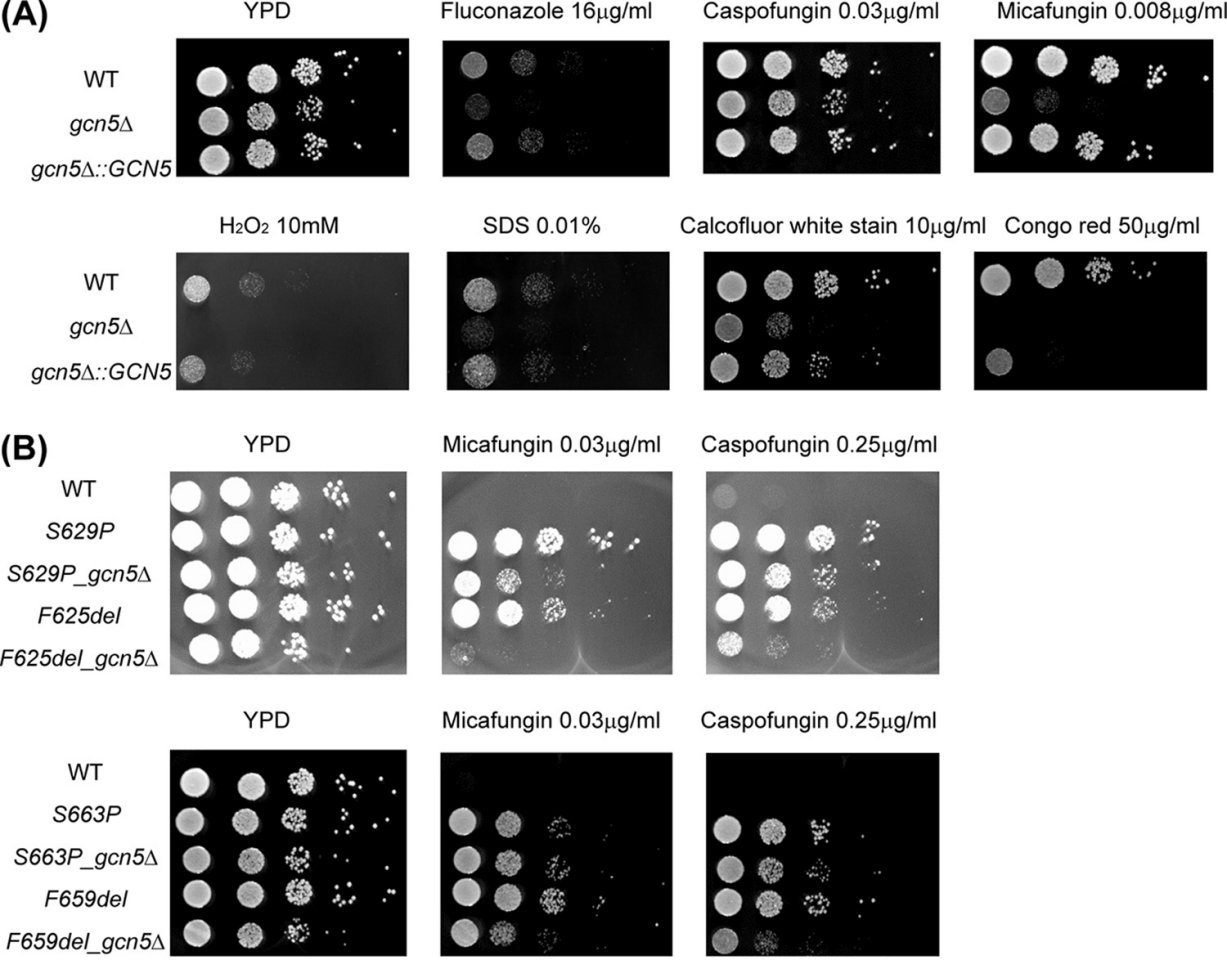
(A) Spotting assay to evaluate stress response changes associated with *GCN5* deletion in C. glabrata. Five microliters of 10-fold serial dilutions of the indicated cells was spotted onto plain YPD plates and YPD plates supplemented with hydrogen peroxide (H_2_O_2_) (10 mM), SDS (0.01%), calcofluor white stain (10 μg/mL), Congo red (50 μg/mL), fluconazole (16 μg/mL), caspofungin (0.03 μg/mL), and micafungin (0.008 μg/mL). Plates were incubated at 37°C for 24 h. (B) Spotting assay with *fks* mutants (Fks1-625delF, Fks1-S629P, Fks2-659delF, and Fks2-S663P) with or without Gcn5 function to assess the impact of *GCN5* on echinocandin resistance. Micafungin was tested at 0.03 μg/mL, and caspofungin was tested at 0.25 μg/mL.

The growth of the WT, the genetically deleted knockout (KO) strain *gcn5*Δ, and reconstituted wild-type strain *gcn5*Δ::*GCN5* was monitored in yeast extract-peptone-dextrose (YPD) broth at 37°C. *gcn5*Δ cells showed slightly slower growth, with an ~10-min-longer doubling time than that of the WT. The growth curve of the *gcn5*Δ::*GCN5* complementary strain is similar to that of the WT (see Fig. S1 in the supplemental material). In limited histone acetylation profiling, acetylated lysine residue 14 of histone 3 (H3K14) and H3K9 in the *gcn5*Δ strain were observed to be decreased to ~46% and 60% of the levels in the WT, respectively (Fig. S2), consistent with the known function of Gcn5. Spotting assays demonstrated that the deletion of *GCN5* conferred increased susceptibility to various cell stress conditions, including oxidative stress, cell wall and cell membrane perturbation, as well as antifungal agents (Fig. 1A). *In vitro* susceptibility testing determined that the MICs of the *gcn5*Δ strain modestly (~2- to 4-fold), yet consistently, decreased for triazole and glucan synthase inhibitor antifungals compared to the WT and complemented strains (Table 1). Interestingly, the *gcn5*Δ strain displayed hypersensitivity to the new antifungal manogepix (formerly APX001A), with an MIC of <0.008 μg/mL. To gain a better understanding of the impact of Gcn5 on echinocandin resistance, we next disrupted *GCN5* from clinically relevant *FKS1* and *FKS2* mutants constructed in the ATCC 2001 background, including Fks1-625delF, Fks1-S629P, Fks2-659delF, and Fks2-S663P. Upon the deletion of *GCN5*, all *FKS* mutants had a 4-fold-lower MIC for micafungin, except for that of the S663P mutant, which decreased only 2-fold (Table 2). This result was largely consistent with what was observed in the spotting assay, where the effect of reduced resistance associated with *GCN5* deletion was seemingly more pronounced in *FKS1* mutants than in *FKS2* mutants (Fig. 1B), suggesting that *GCN5* plays a role in modulating *FKS* expression levels, with differential impacts over *FKS1* and *FKS2* possibly via divergent interactions with other regulators or pathways involved in the regulation network. Given that *FKS2* expression is dependent upon calcineurin signaling (24), we tested whether *GCN5* disruption influences the sensitivity to the calcineurin inhibitor FK506 as well as the effect of the combination of the *GCN5* deletion and FK506 on the cells’ susceptibility to echinocandins. We found that all *GCN5* deletion strains, including both the WT and *FKS* mutants, displayed a 2- to 4-fold increase in susceptibility to FK506, with the *F659del_gcn5*Δ strain demonstrating hypersusceptibility (Table 2). While the addition of FK506 is known to reverse Fks2-mediated echinocandin resistance (24), we found even greater increased susceptibility to micafungin in the presence of FK506 upon *GCN5* deletion. Strikingly, this reversal of resistance was not only relegated to the *FKS2* mutants but also observed with the *FKS1* mutants.

**TABLE 1:** Antifungal susceptibility changes associated with *GCN5* deletion

Antifungal agent	Time of readout (h)	MIC (μg/mL)		
		WT	*gcn5*Δ	*gcn5*Δ::*GCN5*
Itraconazole	24	0.25	0.125	0.25
	48	1	0.25	1

Posaconazole	24	0.5	0.125	0.5
	48	1	0.5	1

Voriconazole	24	0.5	0.25	0.5
	48	1	0.5	1

Fluconazole	24	16	8	16
	48	32	16	32

Anidulafungin	24	0.03	0.015	0.03

Caspofungin	24	0.06	0.06	0.06

Micafungin	24	0.03	0.008	0.015

Manogepix	24	0.064	≤0.008	0.03

Ibrexafungerp	24	0.5	0.25	0.5

**TABLE 2:** Deletion of *GCN5* increases echinocandin susceptibility and reverses Fks1-mediated echinocandin resistance by FK506

Strain	24-h MIC (μg/mL)		
	Micafungin	FK506	Micafungin + FK506 (4 μg/mL)
WT	0.03	32	0.015
*gcn5*Δ	0.008	8	≤0.001
*S629P*	0.5	32	0.25
*S629P_gcn5*Δ	0.125	16	≤0.03
*F625del*	0.5	32	0.125
*F625del_gcn5*Δ	0.125	8	≤0.03
*S663P*	4	32	1
*S663P_gcn5*Δ	2	16	0.5
*F659del*	2	16	0.25
*F659del_gcn5*Δ	0.5	≤0.125	≤0.03

### *GCN5* disruption leads to reduced drug tolerance and resistance development in C. glabrata.

**FIG 2:**
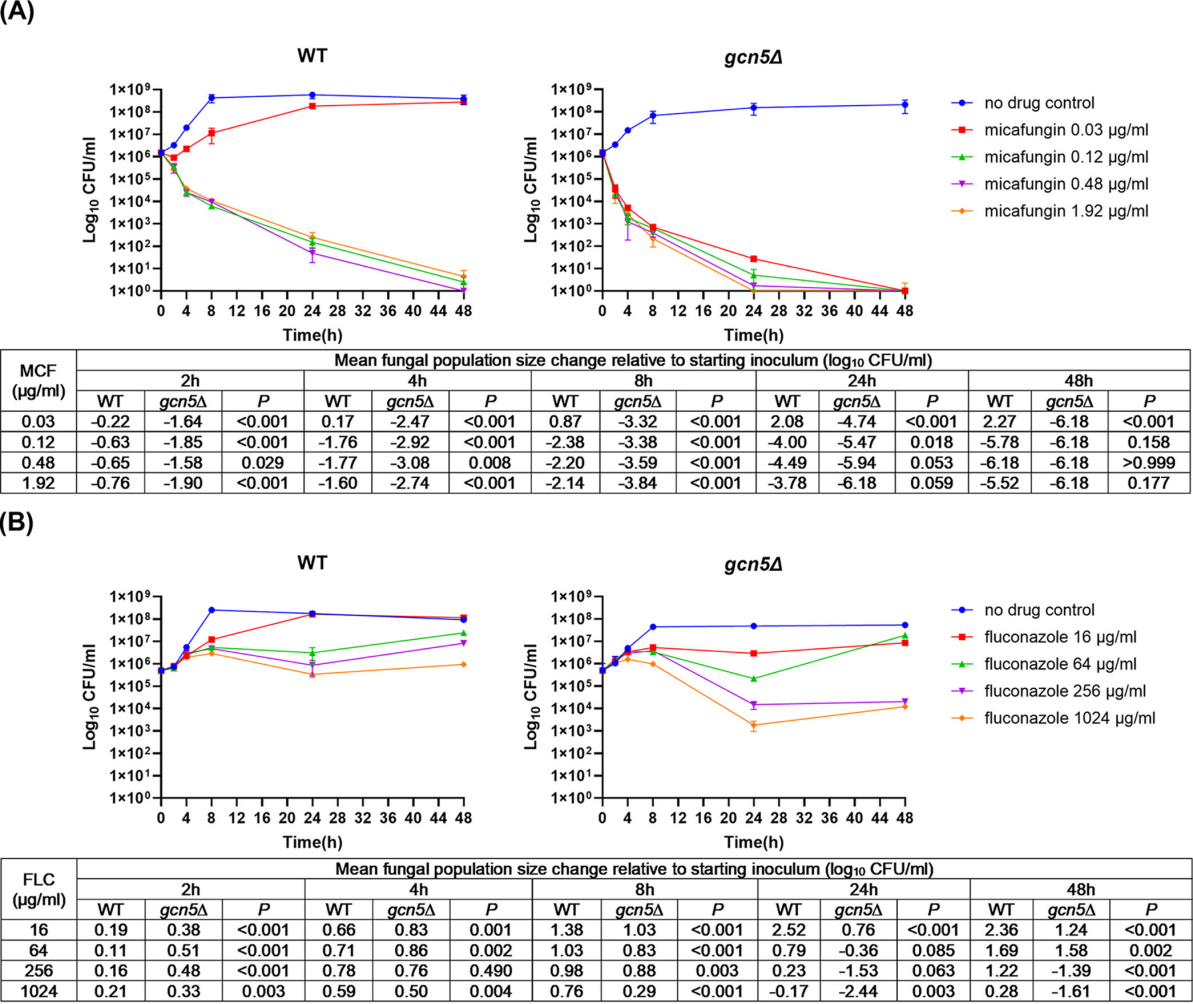
Time-kill curve of micafungin and fluconazole against C. glabrata wild-type (WT) and *gcn5*Δ cells. The cells were incubated at 37°C in RPMI 1640 in the presence or absence of micafungin (MCF) and fluconazole (FLC) at the indicated concentrations. CFU counting was carried out at predetermined time points (0, 2, 4, 8, 24, and 48 h) after drug treatment. (A) Micafungin was significantly more potent against *gcn5*Δ than against WT cells. The 0.03-μg/mL micafungin treatment was fungicidal to *gcn5*Δ but not to WT cells. (B) Fluconazole was more potent against *gcn5*Δ than against WT cells, with significantly enhanced antifungal activity observed with treatment for 24 h or longer at concentrations of 256 and 1,024 μg/mL. Fungal population size change comparisons between WT and *gcn5*Δ cells at each time point are listed underneath the killing curves for micafungin and fluconazole, respectively.

Antifungal tolerance and resistance, impeding effective antifungal therapy and leading to unfavorable clinical outcomes, are two different but relevant phenotypes that fungal pathogens display in response to antifungal agents. Tolerance to fungicidal drugs is defined as the ability of fungal cells to survive at drug concentrations above the MIC (25), and tolerance is considered a key prerequisite for echinocandin resistance in C. glabrata (26, 27). As for fungistatic drugs such as azoles, the concept can be adjusted, and it is reasonable to consider that *Candida* spp. are generally tolerant to azoles (25). To better understand how Gcn5 shapes the antifungal response in C. glabrata, we carried out time-kill studies comparing the survival of the WT and *gcn5*Δ strains in the absence or presence of micafungin or fluconazole, with concentrations ranging from 0.03 to 1.92 μg/mL or from 16 to 1,024 μg/mL, respectively. Enhanced fungicidal activity of micafungin was visualized on killing curves at all levels tested against the *gcn5*Δ strain compared to the WT (Fig. 2A), where a greater colony count reduction was observed with the *gcn5*Δ strain than with the WT at all time points prior to 48 h, and most strikingly, the lowest level of micafungin (0.03 μg/mL) was also fungicidal against the *gcn5*Δ strain yet only fungistatic against the WT. Similarly, the antifungal activity of fluconazole was largely improved upon *GCN5* deletion (Fig. 2B). Despite being considered fungistatic, fluconazole at 1,024 μg/mL resulted in a 2.44-log_10_ CFU/mL reduction of *gcn5*Δ cells after 24 h of treatment.

**FIG 3:**
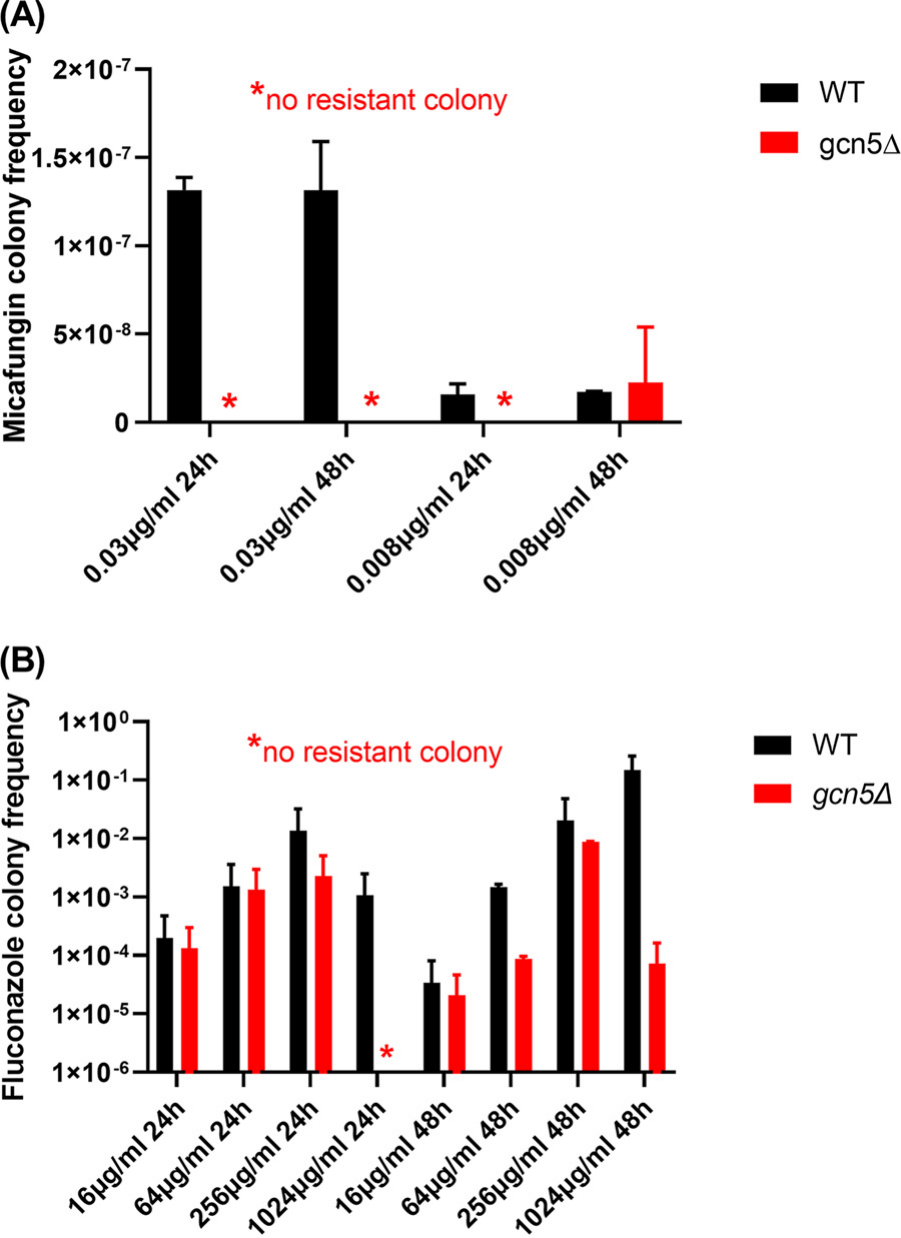
Deleting *GCN5* in C. glabrata leads to reduced resistance development under antifungal pressure. Micafungin resistance (A) and fluconazole resistance (B) were measured in both WT and *gcn5*Δ cells at the 24-h and 48-h time points of the time-kill assay. Cells were plated onto YPD plates containing micafungin (0.25 μg/mL) and fluconazole (512 μg/mL), respectively. The plots show mean resistant colony frequencies ± standard deviations (SD) from ≥3 independent experiments.

To determine the effects of *GCN5* on C. glabrata resistance development, we harvested drug-treated cells at the 24- and 48-h time points of the time-kill study and plated them onto drug-containing plates (0.25 μg/mL micafungin or 512 μg/mL fluconazole) to measure the resistance frequency. Due to the very potent killing of micafungin, resistant colonies were obtained only from WT cells treated with the MIC (0.03 μg/mL) of micafungin, and *gcn5*Δ cells were completely devoid of resistance development within 48 h of exposure to micafungin at all levels tested (Fig. 3A). In the next performed experiment using an extremely low level of micafungin of 8 ng/mL, a low level of resistance (2.23 × 10^−8^) was acquired by *gcn5*Δ cells after 48 h but not 24 h of drug exposure (Fig. 3A and Table 3). Under this condition, WT cells developed micafungin resistance at both 24 and 48 h postexposure at comparable frequencies. Resistant colonies from both the WT and *gcn5*Δ strains were selected for *FKS* sequencing, and all contained Fks2-659delF (Table 3). The *gcn5*Δ strain also exhibited a lower frequency of resistance than that of the WT to fluconazole across all levels tested at both time points (Fig. 3B). Unexpectedly, no *PDR1* mutation was identified from 10 resistant colonies selected from the 256- and 1,024-μg/mL fluconazole treatment groups (Table 3). Together, these data revealed that the deletion of *GCN5* in C. glabrata leads to a significant decrease in antifungal tolerance and resistance development.

**TABLE 3:** Deletion of *GCN5* leads to decreases in frequencies of resistant colonies and resistance-conferring mutations

Antifungal agent	Concn (μg/mL)	Strain	Time (h)	Avg resistant colony frequency ± SD	No. of mutations identified/total no. of colonies sequenced^*a*^	Protein mutation (nucleotide change)
Micafungin	0.03	WT	24	1.32E−07 ± 5.09E−09	5/5	Fks2-659delF (1971_1973delTTC)
			48	1.32E−07 ± 1.94E−08	12/12	Fks2-659delF (1971_1973delTTC)
	0.03	*gcn5*Δ	24	0		
			48	0		
	0.008	WT	24	1.57E−08 ± 4.32E−09	NT	
			48	1.73E−08 ± 1.64E−10	NT	
	0.008	*gcn5*Δ	24	0		
			48	2.23E−08 ± 2.23E−08	4/4	Fks2-659delF (1971_1973delTTC)

Fluconazole	16	WT	24	3.95E−04 ± 7.56E−06	NT	
			48	6.72E−05 ± 1.29E−06	NT	
	16	*gcn5*Δ	24	2.52E−04 ± 1.71E−05	NT	
			48	3.90E−05 ± 2.64E−06	NT	
	64	WT	24	2.99E−03 ± 5.73E−05	NT	
			48	1.60E−03 ± 1.34E−03	NT	
	64	*gcn5*Δ	24	2.84E−03 ± 1.68E−04	NT	
			48	9.33E−05 ± 8.09E−05	NT	
	256	WT	24	2.67E−02 ± 5.12E−04	0/2	No *PDR1* mutation identified
			48	3.98E−02 ± 7.62E−04	0/1	
	256	*gcn5*Δ	24	4.26E−03 ± 2.88E−04	0/1	
			48	8.95E−03 ± 8.77E−03	0/1	
	1,024	WT	24	2.08E−03 ± 3.98E−05	0/1	
			48	2.26E−01 ± 7.22E−02	0/2	
	1,024	*gcn5*Δ	24	0		
			48	1.37E−04 ± 9.28E−06	0/2	

a NT, not tested.

### Perturbed cell wall architecture and reduced adhesion upon *GCN5* deletion revealed by transcriptional profiling.

**FIG 4:**
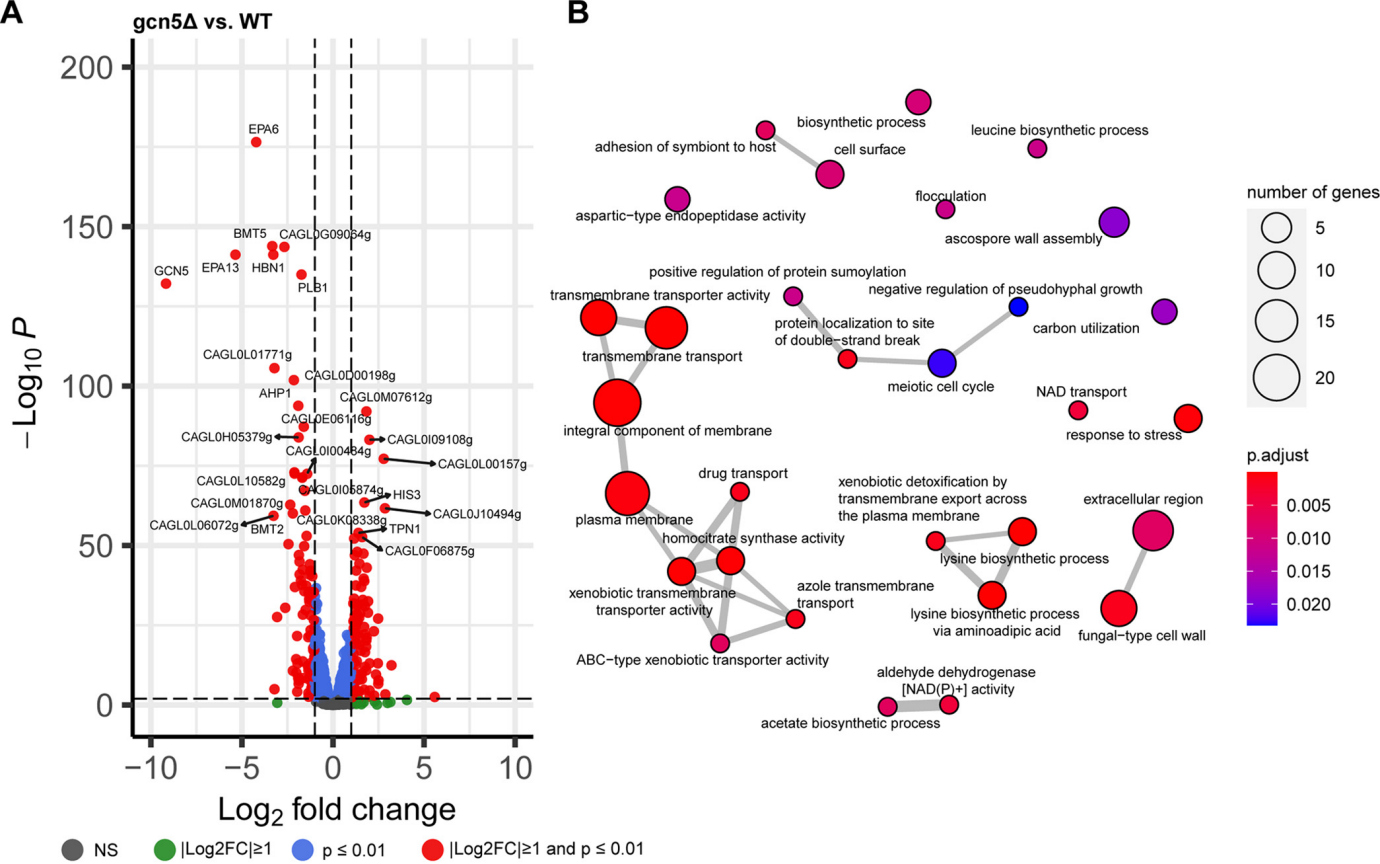
Transcriptional profiling of the *gcn5*Δ strain. Shown are a volcano plot of differentially expressed genes (DEGs) (A) and GO enrichment analysis of DEGs associated with *GCN5* deletion (B). NS, not significant.

**FIG 5:**
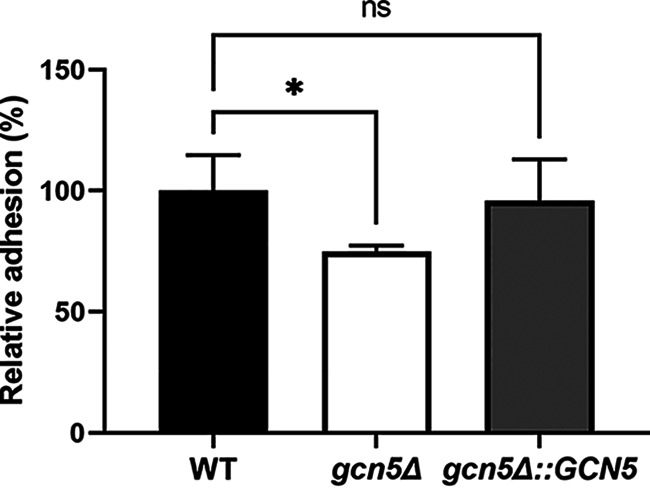
Adhesion to polystyrene after 24 h measured by crystal violet staining. Adherence was normalized to that of the WT. Data represent the means ± SD obtained from 4 technical and 2 biological replicates (*, *P* < 0.05; ns, not significant).

To understand transcriptomic changes associated with *GCN5* disruption in C. glabrata, we performed RNA sequencing (RNA-seq) analysis using WT and *gcn5*Δ cells harvested at logarithmic phase. Totals of 101 and 98 genes were up- and downregulated at least 2-fold with a significant *P* value (*P ≤ *0.01), respectively, in the *gcn5*Δ strain relative to the WT strain (Fig. 4A; Data Set S1). Gene Ontology (GO) enrichment analysis found that genes differentially expressed upon *GCN5* deletion are mainly enriched in cell transmembrane activities and cell wall assembly, as shown in Fig. 4B. Even though the transcriptomic changes seemed to be balanced by the comparable numbers of up- and downregulated genes, ranking of the significant expression differences (less than or equal to −2-fold or greater than or equal to 2-fold with a *P* value of ≤0.01) showed that the top 20 most significantly changed genes upon *GCN5* deletion were predominantly downregulated (Data Set S1). Noticeably, most of the downregulated genes play important roles in cell wall and cell membrane functions, including the cell wall adhesin genes *EPA6* and *EPA13* and the β-mannosyltransferase gene *BMT5*, etc. To validate the RNA-seq findings, a selective set of genes with the most significant changes in transcriptomic profiling were subjected to quantitative real-time reverse transcription-PCR (qRT-PCR) for mRNA expression quantification, and consistent results were observed (Fig. S3). Given that associations between adhesins, particularly *EPA6*, and biofilm formation are well established and that the capacity to strongly adhere to many different surfaces is an important virulence factor of C. glabrata (28, 29), we characterized the adhesion of *gcn5*Δ cells to polystyrene (early stage of biofilm formation) and compared it with those of the parental WT and complemented strains (Fig. 5). Corroborating the transcriptional profiling results, adherence to polystyrene after 24 h of incubation was diminished to ~75% of that of the WT upon the deletion of *GCN5* (*P* = 0.019), whereas it was restored to the WT level when *GCN5* was complemented to the deletion mutant.

To better understand antifungal susceptibility changes associated with *GCN5* deletion, we quantified the expression levels of several key genes known to impact drug tolerance/resistance by qRT-PCR, including *FKS1*, *FKS2*, *CDR1*, *CDR2*, and *CRZ1* (Fig. S3). The lack of *GCN5* resulted in a 1.6-fold drop in *FKS1* expression (*P* = 0.007), accompanied by a possibly compensatory 1.4-fold increase in *FKS2* expression (*P* = 0.09), indicating that *GCN5*-governed transcriptional regulation has a positive modulating effect on *FKS1* but not *FKS2* expression. Given that *FKS1* and *FKS2* in ATCC 2001 are expressed at a roughly 2:1 ratio based on our previous report (30) as well as the current study (data not shown), it is reasonable to envision that the net effect of such opposite expression changes of *FKS1* and *FKS2* in the *gcn5*Δ strain is an overall decrease in the production of β-glucan synthase, explaining the moderately increased echinocandin sensitivity upon *GCN5* deletion. Interestingly, differential regulation was also observed for drug efflux pumps in *gcn5*Δ cells, where *CDR2* expression decreased 3.2-fold (*P* = 0.03) but the *CDR1* mRNA level was not significantly changed compared to those of the WT. Examining the expression of *CRZ1*, a key transcription factor of the calcineurin signaling pathway (31), found that *GCN5* disruption had a modest repression effect (1.5-fold decrease [*P* = 0.18]) on *CRZ1* transcription. Taken together, the genetic ablation of *GCN5* alters the transcriptional landscape, impacting multiple biological processes, primarily cell wall biosynthesis, cell membrane integrity, and transmembrane transport.

### Transcriptomic analysis of WT and *gcn5*Δ cells under antifungal pressure.

**FIG 6:**
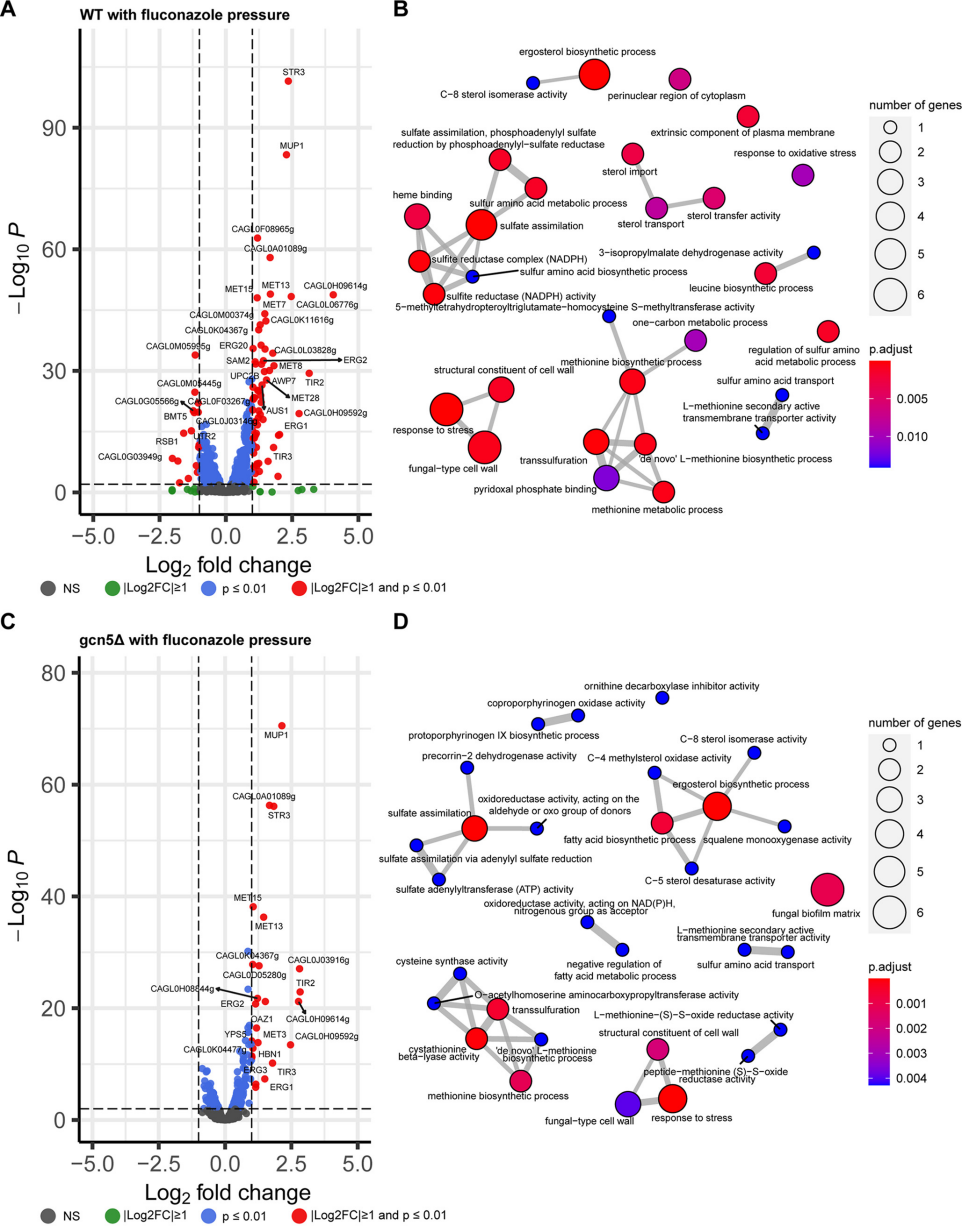
Fluconazole-triggered transcriptional changes. (A and B) Volcano plot of DEGs (A) and GO enrichment analysis of DEGs (B) in WT cells. (C and D) Volcano plot of DEGs (C) and GO enrichment analysis of DEGs (D) in *gcn5*Δ cells.

To uncover the role of Gcn5 in the antifungal response, we subjected both WT and *gcn5*Δ cells to low levels of fluconazole and micafungin, 8 μg/mL and 15 ng/mL, respectively, and analyzed transcriptomic changes induced by the antifungal drug in both cell types after a 2-h exposure. Compared to the no-drug control, fluconazole pressure triggered a globally upregulated transcriptome in WT cells, with 63 genes being significantly upregulated and only 16 being significantly downregulated (Fig. 6A). In comparison, *gcn5*Δ cells displayed a very blunt transcriptional response to the same level of fluconazole stress, with only 24 genes in total being upregulated over 2-fold and no gene being downregulated more than 2-fold (Fig. 6C). A comparison of the two sets of upregulated genes in WT and *gcn5*Δ cells found that most of the significantly induced genes in the *gcn5*Δ strain (18/24) were also upregulated in the WT (Fig. S4). Enriched GO mapping (Fig. 6B and D) also showed that WT and *gcn5*Δ cells shared some similarities in transcriptional responses to fluconazole stress, including oxidation-reduction processes, the stress response, cell wall structure, and ergosterol biosynthesis. To validate the RNA-seq findings, we next selected several genes involved in these biological processes and performed RT-PCR to quantify their expression levels (Fig. S5). Three genes associated with transmembrane transporter activity, *STR3* (putative cystathionine β-lyase), *CAGL0L06776g* (predicted to have DNA-binding transcription factor activity), and *CAGL0L03828g* (orthologs of which have electron transfer activity), were significantly upregulated by ~6- to 15-fold in WT cells upon fluconazole stress but were only slightly induced in *gcn5*Δ cells under the same pressure. Likewise, the expression levels of the putative glycosylphosphatidylinositol (GPI)-linked cell wall protein-encoding genes *CAGL0H09614g* and *AWP7* in fluconazole-treated *gcn5*Δ cells were significantly lower than those in the fluconazole-treated WT strain due to lower increased expression levels in the knockout cells than in the WT under the same drug pressure, consistent with the RNA-seq results. To gain mechanistic insights into the increased sensitivity to azoles observed with *gcn5*Δ cells, we profiled the expression levels of key genes known to influence fluconazole tolerance and resistance, including *ERG11*, *CDR1*, *CDR2*, and *PDR1*, for both strains under fluconazole pressure (Fig. S5). As a result, *ERG11*, *PDR1*, and *CDR2* were all expressed at a significantly lower level in fluconazole-treated *gcn5*Δ cells than in fluconazole-treated WT cells, largely agreeing with the reduced fluconazole tolerability phenotype of the deletion mutant. However, *CDR1* was more highly expressed in *gcn5*Δ than in WT cells upon fluconazole exposure, presumably a compensatory result of the suppressed *CDR2*. Overall, these findings suggest that Gcn5 mediates transcriptome activation involving multiple pathways to resist fluconazole activity; hence, the removal of *GCN5* suppressed an effective transcriptional response, conferring increased sensitivity of knockout cells to fluconazole.

**FIG 7:**
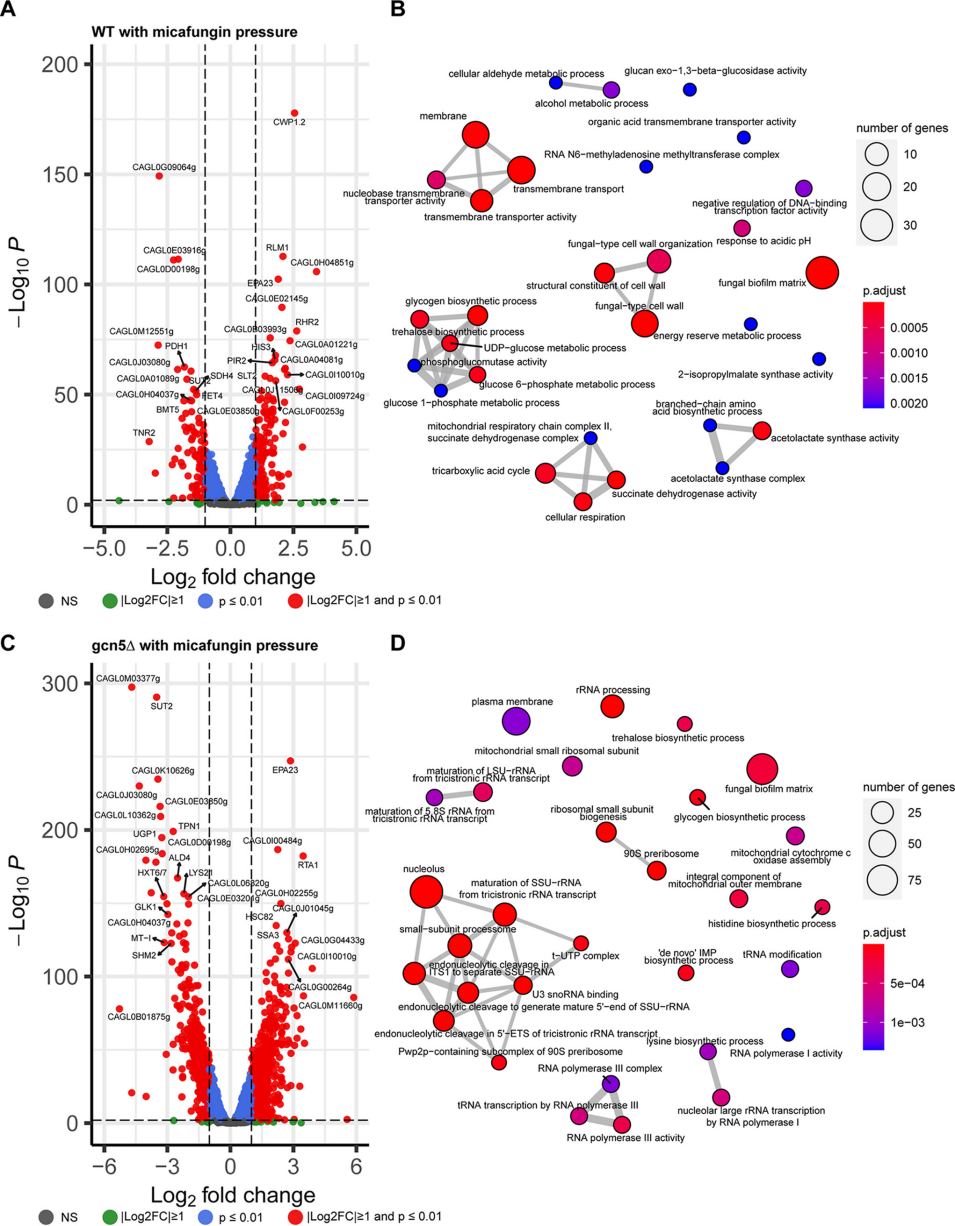
Transcriptional profiling of WT and *gcn5*Δ cells in response to micafungin pressure. (A and B) Volcano plot of DEGs (A) and GO enrichment analysis of DEGs (B) in the WT triggered by micafungin. (C and D) Volcano plot of DEGs (C) and GO enrichment analysis of DEGs (D) in the *gcn5*Δ strain in relation to micafungin treatment. LSU, large subunit; SSU, small subunit; ITS1, internal transcribed spacer 1; ETS, external transcribed spacer.

Unlike fluconazole, the fungicidal drug micafungin exerted much more severe stresses on cells, resulting in a remarkably higher number of genes differentially expressed in both WT and *gcn5*Δ cells than in the corresponding no-drug controls (Fig. 7A and C). Interestingly, the transcriptomic changes occurred at a greater scale in *gcn5*Δ than in WT cells, with ~4 to 5 times more genes altering expression levels over 2-fold in the knockout cells, indicating that cell wall destruction may be a more severe menace for cells lacking *GCN5*; hence, a more extensive transcriptomic adaptation is needed. A Venn diagram (Fig. S6) overlaying the differentially expressed gene sets triggered by micafungin in WT and *gcn5*Δ cells shows that about 60% of the upregulated genes and 2/3 of the downregulated genes in the WT had significant transcriptional changes with the same direction in the *gcn5*Δ strain. However, these overlapping genes account for only <20% of the transcriptomic changes found in the *gcn5*Δ strain. Consistently, enriched GO mapping (Fig. 7B and D) displayed that the cellular pathways involved in the response to micafungin varied in the WT versus the *gcn5*Δ strain. The transcriptional regulation of the WT in the face of micafungin was focused mainly on adjusting the fungal cell wall organization and cell membrane transport activity. Under the same pressure, the *gcn5*Δ strain elicited more extensive transcriptional alterations, a large part of which was to rewire RNA composition, processing, and transcription machinery. The top 10 most significantly changed genes in WT cells in response to micafungin treatment were predominantly upregulated (Data Set S1), whereas downregulation was observed in 8 out of the 10 most significantly changed genes in *gcn5*Δ cells (Data Set S1). To verify these findings, we performed qRT-PCR to quantify the expression levels of a few representative genes (*CAGL0M03377g*, *SUT2*, *CAGL0K10626g*, and *CAGL0I00484g*) (Fig. S7). Not surprisingly, all tested genes had significantly lower expression levels in *gcn5*Δ than in WT cells under micafungin pressure. However, this is not the case for *FKS* genes. The expression level of *FKS2* was 1.78-fold higher in micafungin-treated *gcn5*Δ cells than in micafungin-treated WT cells, although *FKS1* was expressed at comparable levels in both types of cells. These results suggest that *gcn5*Δ cells experienced a higher level of cell wall destruction than WT cells from micafungin exposure, which triggered a more extensive compensatory induction of *FKS* expression.

### Lack of *GCN5* reduces intracellular replication in macrophages.

**FIG 8:**
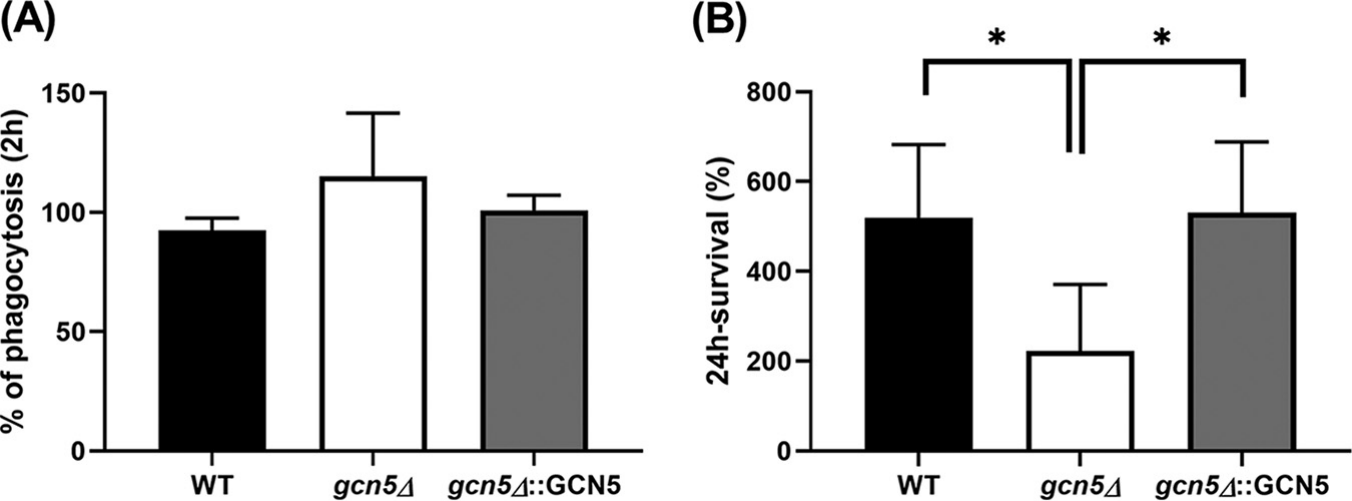
*In vitro* host-pathogen interaction alterations associated with *GCN5* deletion in C. glabrata. (A) Phagocytosis of C. glabrata cells by THP-1 macrophages at 2 h postinfection. (B) The *gcn5*Δ strain had significantly decreased intracellular survival at 24 h postinfection compared to the WT and complemented strains. Data represent the means ± SD from at least 3 independent experiments (*, *P* < 0.05).

To study the role of Gcn5 in host-pathogen interactions, we set up an *in vitro* infection system using the human monocytic cell line THP-1. Phorbol-12 myristate 13-acetate (PMA)-differentiated THP-1 cells were infected with C. glabrata cells at a multiplicity of infection (MOI) of 1:10; thereafter, phagocytosis and intracellular replication were measured at 2 h and 24 h postinfection, respectively. During the initial interaction with macrophages, *gcn5*Δ cells elicited seemingly enhanced phagocytosis, but lacking statistical significance (Fig. 8A), compared to the WT and the complemented strains (115.2% for *gcn5*Δ versus 92.5% for WT and 100.8% for *gcn5*Δ::*GCN5* cells [*P* = 0.18]). However, significantly reduced intracellular replication at 24 h postinfection was observed with *gcn5*Δ cells (Fig. 8B) compared to cells with Gcn5 function (223.1% for *gcn5*Δ versus 519.6% for WT cells [*P* = 0.037]; 223.1% for *gcn5*Δ versus 530.7% for *gcn5*Δ::*GCN5* cells [*P* = 0.023]).

## DISCUSSION

Despite advances achieved in the treatment of fungal infections, antifungal resistance arises at faster paces in multiple human fungal pathogens than that of antifungal drug development, menacing global health. As we deepen our understanding of mechanisms of antifungal resistance, chromatin modification and relevant gene expression regulation start to be recognized as playing pivotal roles in the adaptation of fungal species to antifungal stress, suggesting a potential avenue to tackle antifungal resistance (10, 14). Yet studies in this perspective are highly limited, and a full appreciation of which and how chromatin modifications are involved in the antifungal drug response and resistance is lacking. In light of recent findings of Gcn5 being critical for controlling virulence in C. albicans (20), we aimed to understand how this pleiotropic chromatin modifier shapes the antifungal response and impacts resistance development in C. glabrata, an important human fungal pathogen notorious for the rapid acquisition of antifungal resistance.

C. glabrata lacks hypha formation, a key virulence trait of C. albicans and through which Gcn5 was previously found to impact the stress response and modulate the virulence of C. albicans (19, 20). In our study, deletion of *GCN5* in C. glabrata reduced cell growth in rich medium to only a modest level, but it rendered increased sensitivity of cells to various stressors, including antifungal agents. Using *in vitro* susceptibility testing, we found that the MICs of the *gcn5*Δ strain were consistently decreased modestly by 1 to 2 2-fold dilutions for the azole and echinocandin drugs tested, whereas the hypersensitivity to the new investigational antifungal drug manogepix, which targets GPI anchor biosynthesis maturation, was unexpected. Considering the different targets and mechanisms of action of the drug classes included in our tests, these results suggest that Gcn5 acts as a master regulator coordinating the synthesis of both cell wall and cell membrane structural components. The drastic change in susceptibility to manogepix indicates that Gcn5 deletion-induced cell wall/membrane dysfunction may be synergistic with manogepix blockage of the GPI biosynthesis pathway, which leads to more effective cell killing. In fact, a perturbed cell wall architecture upon *GCN5* deletion was revealed by transcriptional profiling, presenting as markedly downregulated levels of adhesins (*EPA6* and *EPA13*) and β-mannosyltransferase (*BMT5* and *BMT2*) in knockout cells. Such an altered cell wall of the *gcn5*Δ strain may have facilitated the actions of all classes of antifungal agents but largely favored manogepix. Further investigation is warranted to unravel the exact mechanism underlying this phenomenon. Another phenotypic feature stemming from the cell wall changes, particularly the decreased expression of adhesins, associated with *GCN5* deletion was the significantly diminished adherence capacity and biofilm formation of the *gcn5*Δ strain on a polystyrene surface. As adhesion and biofilm formation are well-known virulence factors, this observation is an affirmative testimony to the involvement of Gcn5 in the virulence of C. glabrata. Interestingly, a previous study reported that disrupting *ADA2*, another component of the SAGA complex, conferred mutant hypervirulence, owing at least in part to the highly induced expression of adhesins in the deletion mutant (32). It is worth noting that Gcn5 and Ada2 are interacting partners with distinct functions. Gcn5 mainly serves as a histone acetyltransferase, while Ada2 is more of a transcriptional adaptor (33). The contrasting adhesin expression changing mode from inactivating these two elements individually may reflect the divergent roles of these two proteins in gene regulation.

Given that echinocandins are first-line therapies against invasive *Candida* infections, we attempted to dissect further the relationship between Gcn5 and echinocandin resistance. Therefore, we knocked out *GCN5* from prominent *FKS1* and *FKS2* mutants that are all echinocandin resistant. While all *FKS* mutants demonstrated reduced resistance to echinocandin upon the deletion of *GCN5*, the reversal of resistance was somewhat more pronounced in *FKS1* mutants yet less efficient in *FKS2* mutants, especially the S663P mutant. These results are consistent with *FKS* expression changes found with the *gcn5*Δ strain in the WT background, which showed that the level of the *FKS1* transcript in the *gcn5*Δ strain was less than 60% of the level in the WT strain, accompanied by an ~40% compensatory increase in *FKS2* expression relative to that in the WT. These data imply that the attenuated resistance to echinocandin in *FKS1* mutants upon *GCN5* disruption was at least partially gained through *FKS1* suppression, which outcompeted the compensatory increase in *FKS2* expression. However, the net effect of the restoration of echinocandin susceptibility upon *GCN5* deletion on *FKS2* mutants was more diluted because under the same level of *FKS2* mRNA, mutated Fks2 protein produces resistance, but the WT Fks2 protein does not. Knowing that expression regulation of *FKS2* in C. glabrata is dependent upon calcineurin signaling, we tested the calcineurin inhibitor FK506 for susceptibility in all parental strains (WT and *FKS* mutants) and the corresponding *GCN5* knockout strains. As a result, a 2- to 4-fold decrease in the FK506 MIC was observed in all strains upon *GCN5* deletion, except for the Fks2-659delF mutant being hypersusceptible. Using a sub-MIC of FK506 to block the partial function of Fks2, we found that a greater reversal of micafungin resistance was achieved in all *FKS* mutants, further confirming that Gcn5 mediated *FKS* modulation primarily toward the positive regulation of *FKS1*.

Another key feature associated with *GCN5* deletion was the attenuated resistance acquisition under drug pressure. In the time-kill assay, while WT cells were tolerant to a low level of micafungin (0.03 μg/mL) and developed resistance via *FKS* mutation as quickly as 24 h after exposure, the *gcn5*Δ strain under the same pressure rendered cell death of the majority of the inoculated cell population and was therefore devoid of resistance within 48 h of treatment. Only when micafungin exposure dropped to 0.008 μg/mL did *FKS*-mediated resistance emerge from *gcn5*Δ cells at a frequency comparable to, yet at a pace slower than, that of the WT. Similarly, *gcn5*Δ cells displayed a consistently lower frequency of phenotypic resistance than the WT strain under fluconazole pressure, although we did not identify a *PDR1* mutation from any of the selectively sequenced resistant colonies. These results not only echo the recent findings by Usher and Haynes (22) but also provide an additional layer to understand how *GCN5* impacts echinocandin resistance. To understand how *GCN5* regulates the cell response to these two most common antifungal classes, we profiled the transcriptomes of both the WT and *gcn5*Δ strains in the presence and absence of modest levels of fluconazole and micafungin. As expected, we found that a 2-h exposure to a sub-MIC of fluconazole led to a largely induced transcriptome in the WT strain, particularly involving membrane, transmembrane transporter activity, and fungal cell wall organization pathways. However, the *gcn5*Δ strain displayed a markedly rigid transcriptomic profile in response to fluconazole exposure, with only 22 genes in total being upregulated over 2-fold. Notably, even though the WT and *gcn5*Δ strains showed similar patterns of differential expression upon fluconazole exposure, the *gcn5*Δ strain tended to be less responsive to cell damage caused by fluconazole; therefore, the expression changes of the *gcn5*Δ strain were mostly smaller than those of the WT. These results suggest that Gcn5 in C. glabrata plays a role in prompting swift cell defense and activating effective adaptation to fluconazole. Intriguingly, when drug exposure was switched to micafungin, a highly disturbed transcriptome of the *gcn5*Δ strain was observed, with approximately 4 times more genes being up- or downregulated >2-fold than in the WT. Enriched GO mapping also showed highly divergent transcriptomic responses in *gcn5*Δ and WT cells. A considerable number of differentially expressed genes in *gcn5*Δ cells were relevant to ribosome biogenesis, which may be a snapshot of the cells’ last line of defense against life-threatening stress from micafungin treatment. In comparison, transcriptomic changes in WT cells were more focused on upregulating cell wall integrity pathway genes as well as transmembrane transporter activity, suggesting that the cells were operating a concerted machinery to resist and repair cell wall damage induced by micafungin. This observation is also consistent with the most recently published study investigating transcriptomic alterations in C. glabrata cells surviving micafungin treatment (26).

As a successful human pathogen, C. glabrata is highly adapted to interaction with host cells and the immune response. Macrophages are professional phagocytes that act as part of the innate immune system of the host, contributing to antifungal defense via phagocytosis and clearance of invading fungal pathogens (34). Phagocytosis of C. glabrata starts with pattern recognition of fungal cell wall components such as β-glucan and mannan by the C-type lectin receptors dectin-1 and dectin-2 on macrophages (35, 36). Hence, a disturbed cell wall architecture is deemed to alter C. glabrata-macrophage interactions, as observed in previous studies involving deletion mutants lacking cell surface-associated aspartyl proteases and those with defects in protein glycosylation (37, 38). In view of both phenotypic and transcriptomic data suggestive of an altered cell wall composition upon *GCN5* deletion, we attempted to determine whether the loss of Gcn5 has an impact on host-pathogen interactions using *in vitro*-differentiated THP-1 macrophages. Indeed, the *gcn5*Δ strain demonstrated significantly reduced intracellular survival compared to the WT, while the phagocytosis of mutant cells by macrophages was slightly more efficient. The production of toxic reactive oxygen species (ROS) is one central aspect of the macrophage antimicrobial response. Despite evidence that C. glabrata possesses robust and redundant antioxidant systems conferring high-level resistance to oxidative stress, ROS production in macrophages at least partially contributes to the intracellular killing of this fungus (39). Taking the phenotypic testing results into consideration, the reduced survival of *gcn5*Δ cells within THP-1 macrophages is somewhat expected and may be due partly to the increased sensitivity of the deletion mutant to oxidative stress and/or other stresses encountered in the macrophage internal milieu. The fact that *gcn5*Δ cells were phagocytosed at a slightly higher rate than WT cells suggests that cell wall disturbance, presumably improper construction and/or assembly of GPI-anchored proteins, especially adhesins (Epa6 and Epa13), may have resulted in a modestly increased exposure of the skeletal components to macrophages. Previous studies have shown that Epa6 is a significant virulence factor of C. glabrata that contributes strongly to both biofilm formation and adherence to epithelial cells to establish experimental urinary tract infection (28, 40). In these backgrounds, our results suggest that blocking the function of Gcn5 may be a potential way to not only attenuate the virulence of C. glabrata but also facilitate host cells clearing infections, although more in-depth studies are needed.

Taken together, the results of our study show that the histone acetyltransferase Gcn5 plays a critical role in modulating the virulence of C. glabrata and regulating its response to antifungal pressure and host defense. Despite great interest raised in the past in using chromatin modification targets to aid in antifungal discovery (14), there is a lack of appreciation for how epigenetic mechanisms, such as histone acetylation, are involved in the antifungal response and resistance development. The findings of the present study provide insights into this understudied topic and demonstrate the possibility of limiting resistance in C. glabrata as well as enhancing the efficacy of existing antifungal therapy and promoting host defense by inactivating Gcn5 function. In theory, chemical inhibition should mimic some of the phenotypes obtained by genetic ablation. Therefore, future studies exploring and evaluating specific Gcn5 inhibitors as a potential adjunct to existing antifungal therapy to improve clinical outcomes are warranted.

## MATERIALS AND METHODS

### Strains and growth conditions.

C. glabrata strain ATCC 2001 was obtained from the American Type Culture Collection (Manassas, VA). *FKS1* and *FKS2* mutants (Fks1-625delF, Fks1-S629P, Fks2-659delF, and Fks2-S663P) were constructed in the ATCC 2001 background (see Table S1 in the supplemental material). All C. glabrata strains were grown at 37°C in yeast extract-peptone-dextrose (YPD) medium (1% yeast extract, 2% peptone, and 2% dextrose) with shaking at 150 rpm.

### *GCN5* disruption and complementation.

To disrupt *GCN5*, *NAT1* was amplified from plasmid pCN-PDC1 (41) with primers that contained overhangs homologous to the up- and downstream regions of C. glabrata *GCN5* (Table S1). The purified deletion cassette, *GCN5* guide RNA (designed online at https://chopchop.cbu.uib.no/), and the Alt-R CRISPR-Cas9 system (Integrated DNA Technologies, Inc.) were transformed into competent WT (or *FKS* mutant) cells, as previously described (30). Transformants that grew on YPD plates supplemented with 100 μg/mL nourseothricin were PCR screened and sequenced to confirm the correct deletion. To complement the *gcn5*Δ mutant, we PCR amplified the coding region of *GCN5* from ATCC 2001 genomic DNA (Table S1). Guide RNA targeting the *NAT1* region was designed using the online software CHOPCHOP. The purified *GCN5* repair cassette, *NAT1* guide RNA, and the Alt-R CRISPR-Cas9 system were transformed together into competent deletion mutant cells. Transformants grown on plain YPD plates were replica plated onto nourseothricin-containing YPD plates, and those that grew only on plain YPD plates (nourseothricin sensitive) were further PCR screened and sequenced to confirm successful complementation. All primers used for this procedure are listed in Table S1.

### Growth curve and doubling time measurement.

Cultures of each C. glabrata strain grown overnight were diluted to an optical density at 600 nm (OD_600_) of 0.1 with fresh YPD medium. The absorbance was recorded every 15 min for 12 h by a microplate spectrophotometer (VersaMax enzyme-linked immunosorbent assay [ELISA] microplate reader with SoftMax Pro software; Molecular Devices). Each strain was tested in triplicate, and OD_600_ values were plotted versus time. The doubling times were calculated as previously described (42).

### Western blotting.

Gcn5 is known to acetylate multiple histone lysine residues, primarily lysine residue 14 of histone 3 (H3K14) as well as H3K9, H3K18, H3K23, H3K27, H3K36, and other additional lysine residues found in histones H4 and H2B (43, 44). To validate epigenetic changes associated with *GCN5* disruption, we employed Western blot analysis to compare histone acetylation levels in WT, *gcn5*Δ, and *gcn5*Δ::*GCN5* cells using H3K14 and H3K9 as representative targets. Whole-cell lysates were prepared by trichloroacetic acid (TCA) precipitation. Briefly, cell pellets from cultures grown overnight were resuspended in 20% TCA, disrupted by bead beating, and washed twice with 5% TCA. Protein was pelleted and resuspended in sodium dodecyl sulfate-polyacrylamide gel electrophoresis (SDS-PAGE) loading buffer, followed by incubation at 95°C for 5 min and centrifugation prior to loading onto 16% acrylamide gels. Primary antibodies for immunoblotting were obtained commercially, including histone H3 antibody (catalog no. 4499; Cell Signaling Technology), acetyl-histone H3(Lys9) (H3K9Ac) antibody (catalog no. 9649; Cell Signaling Technology), acetyl-histone H3(Lys14) (H3K14Ac) antibody (catalog no. 7627; Cell Signaling Technology), and β-actin antibody (catalog no. PA5-85271; Thermo Fisher Scientific). The target protein was visualized with Novex ECL chemiluminescent substrates (Thermo Fisher Scientific) according to the manufacturer’s instructions, and band intensities were determined using ImageJ software (https://imagej.nih.gov/ij/).

### *In vitro* susceptibility testing.

Antifungal susceptibility testing was performed at least in duplicate for each strain according to CLSI guidelines (45). Micafungin (Astellas, Deerfield, IL), caspofungin (Merck, Rahway, NJ), anidulafungin (Pfizer, New York, NY), fluconazole (LKT Laboratories, Saint Paul, MN), voriconazole (Pfizer, New York, NY), itraconazole (Sigma, St. Louis, MO), posaconazole (Sigma, St. Louis, MO), FK506 (Tecoland, Edison, NJ), ibrexafungerp (Scynexis, Inc., Jersey City, NJ), and manogepix (formerly Amplyx Pharmaceuticals, Inc., San Diego, CA) were dissolved and diluted according to CLSI standards (45). To determine the functionality of Fks1, we also performed micafungin susceptibility testing in the presence of FK506 at 4 μg/mL.

### Spotting assay.

C. glabrata cells grown overnight were washed twice with phosphate-buffered saline (PBS) and adjusted to ~5 × 10^5^ CFU/mL in PBS. Equal volumes (5 μL) of 10-fold serial dilutions of each strain were spotted onto YPD plates containing various cell stress agents, including 10 mM H_2_O_2_, 0.01% SDS, calcofluor white stain (10 μg/mL), and Congo red (50 μg/mL), as well as antifungal agents consisting of fluconazole (16 μg/mL), micafungin (0.008 and 0.03 μg/mL), and caspofungin (0.03 and 0.25 μg/mL). Colony growth was compared with that on the plain YPD control plate after 24 h of incubation at 37°C.

### Time-kill assay.

Time-kill studies were performed to compare the tolerances of the WT (ATCC 2001) strain and the *gcn5*Δ strain to micafungin and fluconazole, according to a procedure described previously, with minor modifications (46). Each drug at concentrations of 1×, 4×, 16×, and 64× WT MIC as well as a no-drug control were included in the evaluation. RPMI 1640 buffered with morpholinepropanesulfonic acid (MOPS) was the growth medium. The starting inoculum of each strain was prepared in a total volume of 5 mL at 1 × 10^6^ CFU/mL for the experiment involving micafungin and 5 × 10^5^ CFU/mL for that involving fluconazole. All samples were incubated at 37°C with shaking at 150 rpm, and a 15-μL aliquot was taken from each sample at 0, 2, 4, 8, 24, and 48 h. The removed aliquots were pelleted and washed in PBS, and proper dilutions were then prepared, plated in duplicate onto YPD plates, and incubated at 37°C for 24 h to determine the colony counts. The frequency of resistant colonies was measured at 24 and 48 h for all samples in duplicate, using YPD plates containing 0.25 μg/mL micafungin or 512 μg/mL fluconazole. Hot spot 1 and 2 regions of *FKS1* and *FKS2* were sequenced for micafungin-resistant colonies, and *PDR1* sequencing was performed for fluconazole-resistant colonies, using primers listed in Table S1.

### RNA isolation and RNA-seq analysis.

Cultures of the WT and *gcn5*Δ strains grown overnight were inoculated in liquid YPD medium at an initial OD_600_ of 0.2 and grown at 37°C with shaking to an OD_600_ of 0.6. Cells were then treated with fluconazole (8 μg/mL) or micafungin (15 ng/mL) for 2 h. Untreated WT and *gcn5*Δ cells were grown in parallel. Cells were harvested at the end of the 2-h treatment and subjected to RNA extraction, with two biological replicates for each strain/treatment group. Total RNA was extracted from each sample according to a protocol described previously (30), and RNA samples were stored at −80°C until shipping to Genewiz (South Plainfield, NJ) for RNA sequencing. The RNA-seq data were processed using the Illumina NovaSeq platform with 150-bp paired-end reads. The read data quality was examined using FastQC v.0.11.9 (https://www.bioinformatics.babraham.ac.uk/projects/fastqc/), and reads were trimmed to remove low-quality bases (average quality per base of <20) using Trimmomatic v.0.39 (47). The filtered reads were then aligned to the Candida glabrata CBS138/ATCC 2001 genome (www.candidagenome.org) using HISAT 2.2.1 (48), and the raw read counts were obtained using HTSeq v.0.12.3 (49). Differentially expressed genes (DEGs), defined as genes with an absolute log_2_ fold change [|log_2_FoldChange|] of ≥1 and adjusted *P* values of ≤0.05, across different groups were estimated using DEseq2 v.1.34.0 (50) in R 4.0.2. Gene Ontology (GO) annotation was downloaded from the *Candida* Genome Database (CGD) (www.candidagenome.org), and enrichment analysis was done using clusterProfiler 4.0 (51) in R.

### Quantitative real-time reverse-transcription-PCR.

To verify the RNA-seq results, the expression levels of representative genes selected from each comparison were measured by reverse transcription-PCR (RT-PCR) using one-step SYBR PrimeScript RT-PCR kit II (TaKaRa, Shiga, Japan). Reaction mixtures were run on an Mx3005P quantitative PCR (qPCR) system (Agilent Technologies, CA, USA) and contained 10 ng RNA sample, 0.4 μM each primer (Table S1), 12.5 μL 2× one-step SYBR RT-PCR buffer, and 1 μL PrimeScript one-step enzyme mix 2 in a volume of 25 μL. Thermal cycling conditions were as follows: RT at 42°C for 5 min; PCR cycling with an initial denaturation step at 95°C for 10 s, followed by 40 cycles of denaturation at 95°C for 5 s and annealing and elongation at 60°C for 20 s; and a post-PCR melting-curve analysis at 95°C for 5 s and 60°C for 1 min and then increasing to 95°C with a ramp rate of 0.5°C/s (30). Each experiment was carried out in duplicate, and negative controls were included in each run. The *PGK1* gene was used as a reference gene to normalize the data (52). Relative quantification of gene expression was performed using the 2^−ΔΔ^*^CT^* method (53). The fold changes were determined as the mean normalized expression of mutant or treated samples relative to the mean normalized expression of the untreated ATCC 2001 control. Statistical analysis of gene expression was carried out using GraphPad Prism software, and a *P* value of <0.05 was considered significant.

### Adhesion assay.

C. glabrata cells grown overnight in liquid YPD medium were adjusted to an OD_600_ of 1.0 in fresh YPD broth, 200 μL of which was inoculated into a 96-well microtiter plate. After 24 h of incubation at 37°C without shaking in a humid environment, unattached cells were removed by gentle washing with distilled water three times. Plates were air dried, 100 μL of 0.1% (wt/vol) crystal violet was then added to each well, and the plates were incubated at 37°C for 30 min, followed by gentle washing with distilled water and air drying. Finally, 200 μL of 33% glacial acetic acid was added to each well, and adhesion was quantified by measuring the OD_595_ using a plate reader (Infinite Pro; Tecan). All strains (WT, *gcn5*Δ, and *gcn5*Δ::*GCN5*) were tested in parallel in four technical and two biological replicates.

### THP-1 macrophage infection.

The human monocyte cell line THP-1 (ATCC TIB202) was cultured in RPMI 1640 medium supplemented with 10% heat-inactivated fetal bovine serum (FBS) at 37°C under 5% CO_2_. The THP-1 macrophage infection process was performed as described previously by Rasheed et al., with minor modifications (37). Briefly, 24-well plates were seeded with 1 × 10^6^ THP-1 cells, and THP-1 cells were differentiated into macrophages for 16 h in the presence of 16 nM phorbol-12 myristate 13-acetate (PMA), followed by a 12-h recovery. On the day of infection, 50 μL of a suspension of 1 × 10^6^ CFU/mL of C. glabrata cells (WT, *gcn5*Δ, and *gcn5*Δ::*GCN5*) was added to each well of the THP-1 cells to obtain a multiplicity of infection (MOI) of 1:10 (fungi to macrophages) and incubated at 37°C with 5% CO_2_. After 2 and 24 h of coculture, macrophages were washed three times with prewarmed sterile PBS to remove nonphagocytized extracellular C. glabrata cells. Macrophages were then lysed in water, and 100 μL of the properly diluted lysate was plated onto a YPD plate to determine the intracellular CFU counts. The phagocytosis rate for each strain was measured using the 2-h CFU counts divided by the CFU of the inoculum. The intracellular replication of C. glabrata for each strain was calculated by dividing the CFU at the 24-h time point by those at 2 h.

### Data availability.

Raw RNA-seq data have been deposited at the Gene Expression Omnibus (accession no. GSE194310).
