# Supplementary material for: Histone Acetylation Regulator Gcn5 Mediates Drug Resistance and Virulence of Candida glabrata
Source: Microbiol Spectr. 2022 Jun 6;10(3):e00963-22. doi: 10.1128/spectrum.00963-22 (PMC9241792; doi:10.1128/spectrum.00963-22)
Supplement: Supplemental file 1 — Supplemental material. Download spectrum.00963-22-s0001.pdf, PDF file, 0.5 MB [file spectrum.00963-22-s0001.pdf]

Histone Acetylation Regulator Gcn5 Mediates Drug Resistance and Virulence of *Candida glabrata*

Shuying Yu[1,2,3], Padmaja Paderu[1], Annie Lee[1], Sami Eirekat[4], Kelley Healey[4], Liang Chen[1,5], David S. Perlin[1,5], Yanan Zhao[1,5]*

[1]Center for Discovery and Innovation, Hackensack Meridian Health, Nutley, New Jersey, USA.
[2]Department of Clinical Laboratory, Peking Union Medical College Hospital, Chinese Academy of Medical Sciences, Beijing, P. R. China.
[3]Beijing Key Laboratory for Mechanisms Research and Precision Diagnosis of Invasive Fungal Diseases (BZ0447), Beijing, China.
[4]Department of Biology, William Paterson University, Wayne, New Jersey, USA.
[5]Department of Medical Sciences, Hackensack Meridian School of Medicine, Nutley, New Jersey, USA.
[6]Department of Microbiology and Immunology, Georgetown University School of Medicine, Washington, DC 20057

* Address correspondence to Yanan Zhao, Ph.D. yanan.zhao@hmh-cdi.org, phone: 201-880-3503

SUPPLEMENTARY MATERIALS

***FKS* mutant construction methods**

FKS mutant strains were constructed as described in Healey et al., 2020 (1). Briefly, Fks1-625delF, Fks1-S629P, Fks2-659delF, and Fks2-S663P were generated in strain ATCC 2001 through transformation of a purified PCR product. Desired mutations were PCR-amplified along with regions flanking the *FKS1* or *FKS2* hotspot 1 region (approximately 400 bp) from mutant isolates (see **Table S1** for primers). Transformants were selected on low levels of caspofungin- (0.3 µg/mL) or micafungin- (0.03 µg/mL) containing YPD agar medium. All *FKS1* and *FKS2* hotspots were sequenced in each transformant to confirm the expected mutation was present and all other amino acids remained unaltered.

Figure S1. Growth curves of *Candida glabrata* wild type (WT), *gcn5*Δ and *gcn5*Δ::*GCN5* strains in YPD media.

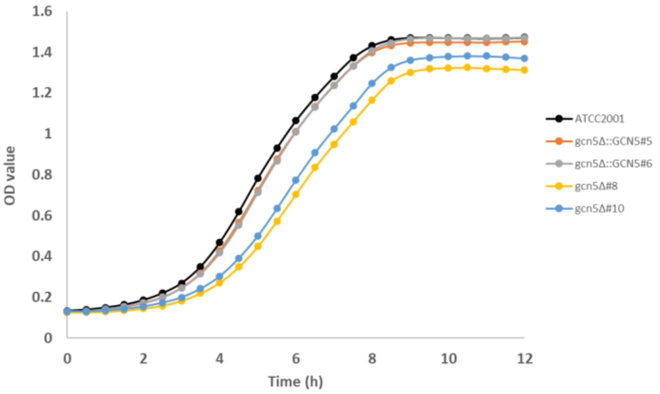

Figure S2. (A) Immunoblots showed visibly decreased abundance of both H3K9Ac and H3K14Ac in *gcn5*Δ, compared to WT and complemented strains. (B) Acetylation level comparison in WT, *gcn5*Δ, and *gcn5*Δ::*GCN5*. Signals of H3K9Ac and H3K14Ac were normalized to that of H3 in corresponding strain. Acetylated H3K9 and H3K14 in *gcn5*Δ decreased to 60% and 46% of that in WT, respectively. Representative blots from two independent experiments and mean ratios ± SD are shown.

(A)

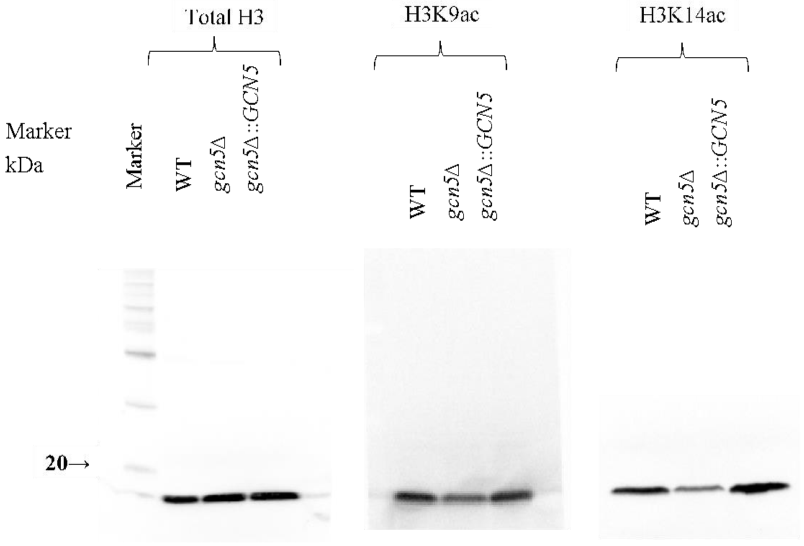

(B)

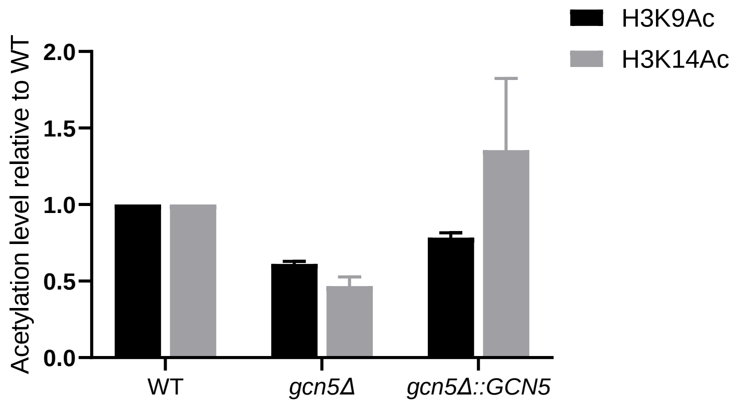

Figure S3. qRT-PCR verification of representative DEGs in *gcn5*Δ cells. Expression was normalized to reference gene *PGK1* and shown as a fold change compared to WT. Plots represent the mean ± SD from ≥ 3 independent experiments (**P*<0.05, ***P*<0.01, ****P*<0.001).

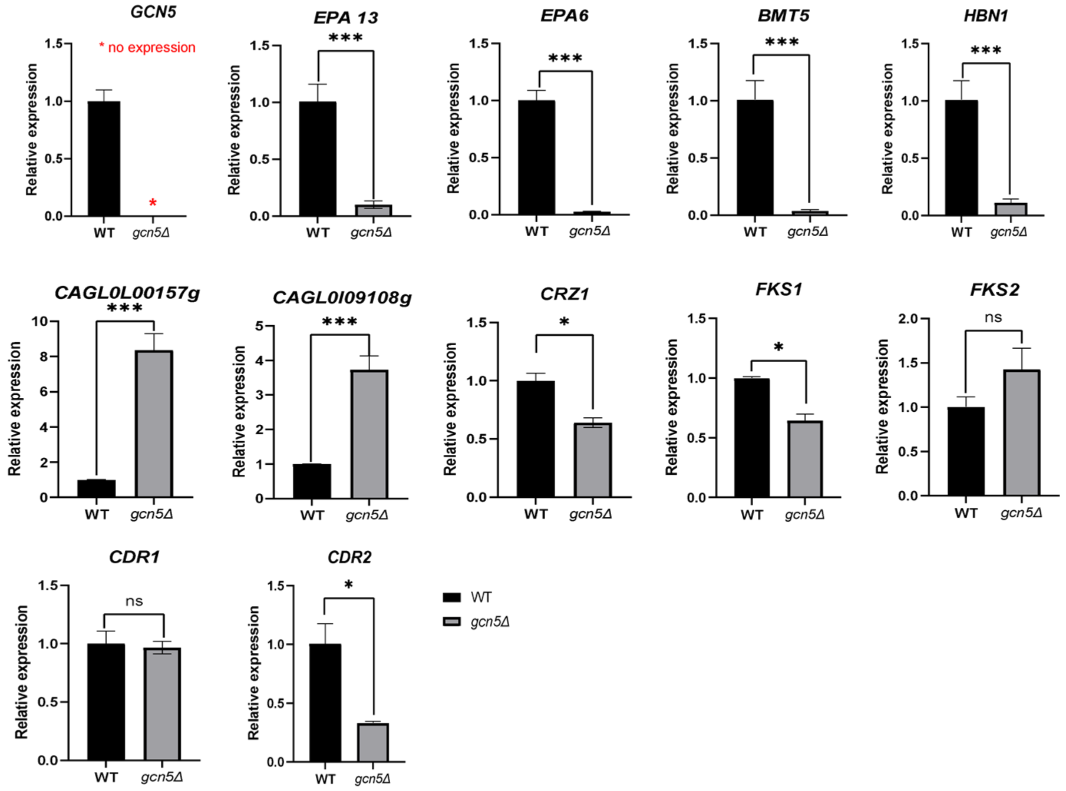

Figure S4. Venn diagram of upregulated gene sets in WT and *gcn5*Δ associated with fluconazole treatment.

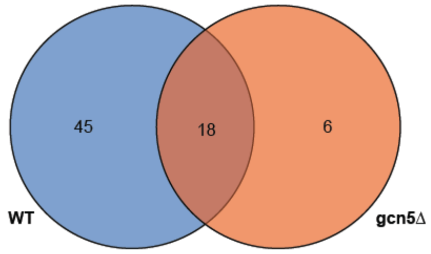

Figure S5. qRT-PCR verification of representative DEGs in WT and *gcn5*Δ cells in response to fluconazole (FLC) pressure. Expression was normalized to reference gene *PGK1* and shown as a fold change compared to WT no drug control (NDC). Plots represent the mean ± SD from ≥ 3 independent experiments (**P* <0.05, ***P*<0.01, ****P*<0.001).

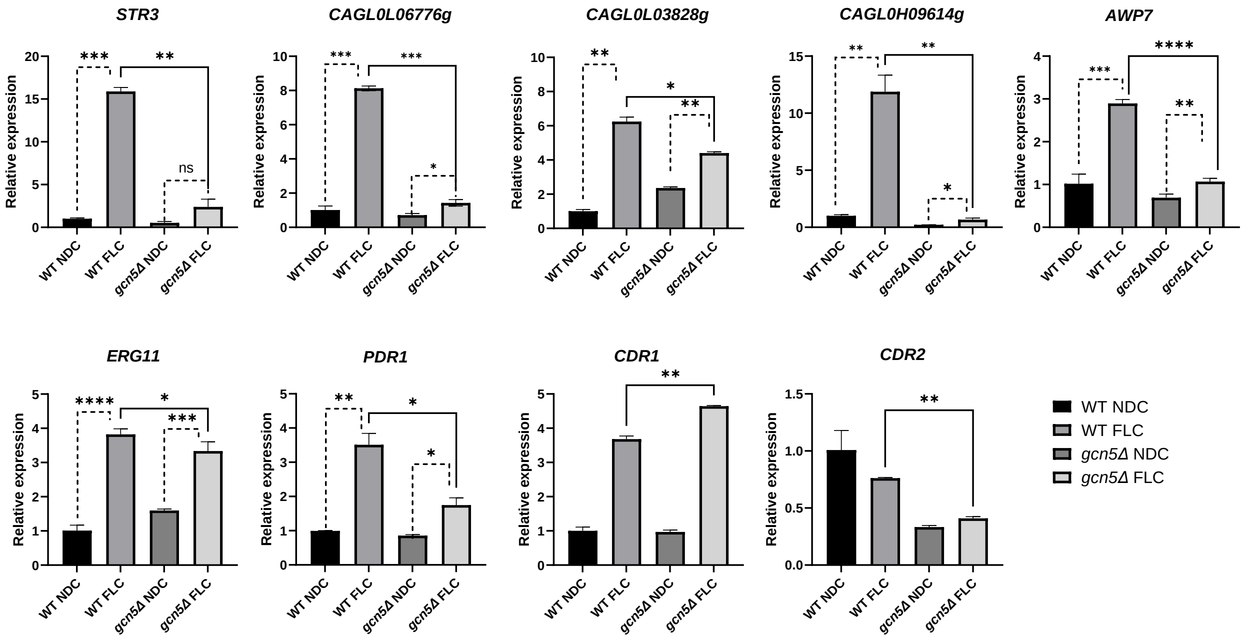

Figure S6. Venn diagram of micafungin triggered up- and down-regulated gene sets in WT and *gcn5*Δ cells.

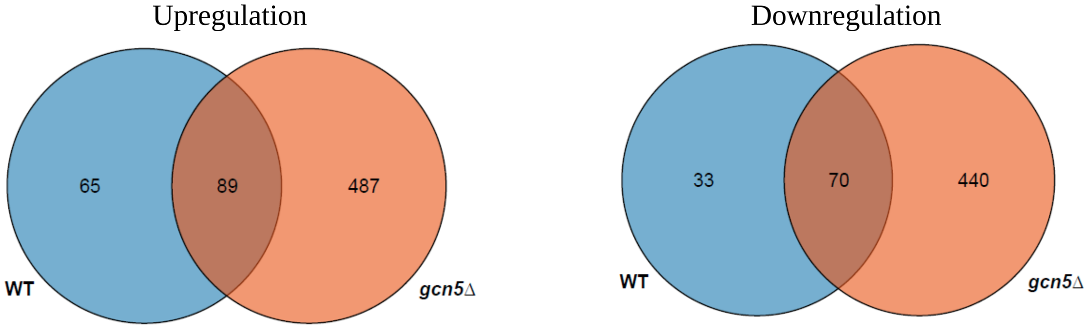

Figure S7. qRT-PCR verification of representative DEGs in WT and *gcn5*Δ cells triggered by micafungin (MCF) treatment. Expression was normalized to reference gene *PGK1* and shown as a fold change compared to WT no drug control (NDC). Plots represent the mean ± SD from ≥ 3 independent experiments (**P* <0.05, ***P*<0.01, ****P*<0.001, ns denotes for no statistical significance).

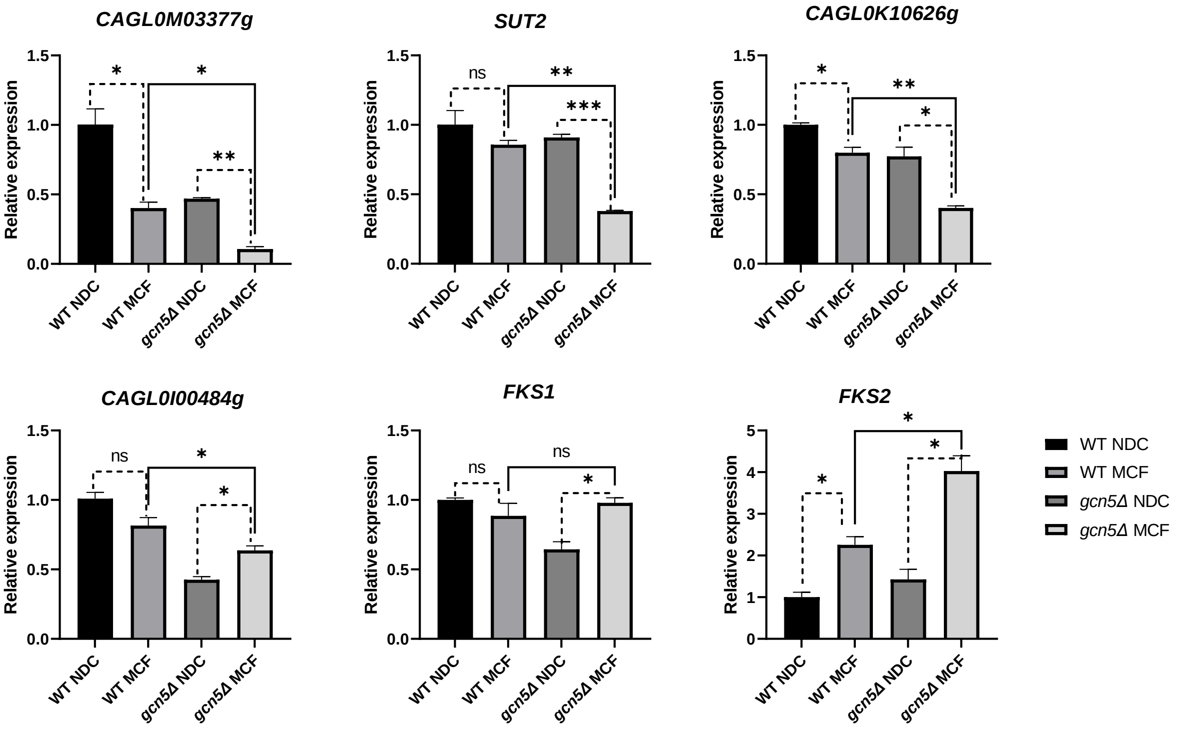

Table S1. Sequences of primers and oligos used in this study

Oligo	Sequence (5'-3')	Application
GCN5-F364	CCATTACACAATGTTTTACCCG	*GCN5* upstream PCR
pRS_GCN5_R-67	CTCAGTACAATCTGCTCTGATGCCGGCAA CTCAAACTGTTCGCCTCG	*GCN5* upstream PCR
CYC1_GCN5_F1757	AGTTATGTCACGCTTACATTCACGCAGTAT GTAGAAACACGGTAAATAGAC	*GCN5* downstream PCR
GCN5-R2071	GGGTAAAACACGGCAACAAA	*GCN5* downstream PCR
pRS-F	CGGCATCAGAGCAGATTGTA	Amplification of *NAT* cassette from pCN-PDC1
CYC1-R	GCGTGAATGTAAGCGTGAC	Amplification of *NAT* cassette from pCN-PDC1
TEFp263F	TTCGATGACCTCCCATTGAT	*GCN5* knockout validation sequencing
NAT297R	GTACGAGACGACCACGAAGC	*GCN5* knockout validation sequencing
Gcn5-crRNA	GAGAGGUGAACAAUCCACCGGUUUUAGA GCUAUGCU	CRISPR crRNA, N20 sequence underlined
NAT-crRNA	CUGGACACCGCCCUGUACGAGUUUUAGA GCUAUGCU	CRISPR crRNA, N20 sequence underlined
CgFKS1c1757F	ACGTCGCTTCTCAAACCTTC	*FKS1* mutant construction, hotspot 1 PCR
CgFKS1c2225R	GCGTTCCAGACTTGGGAAAT	*FKS1* mutant construction, hotspot 1 PCR/sequence
CgFKS1c1674F	GTTGCAGTCGCTACATTGCTA	screen/sequence
CgFKS1c3918F	CGCTCTTGCACACGAATCTA	*FKS1* mutant construction, hotspot 2 PCR
CgFKS1c4225R	CACCACCAACAGTCAAATCG	*FKS1* mutant construction, hotspot 2 PCR/sequence
CgFKS2c1790F	CGATTATGCCATTAGGTGGTC	*FKS2* mutant construction, hotspot 1 PCR
CgFKS2c2165R	CCAACAGAGAAGACAGTGTTGA	*FKS2* mutant construction, hotspot 1 PCR/sequence
CgFKS2c1419F	GGATTATGCACGTTTCCGTC	screen/sequence
CgFKS2c3930F	GCATCCTGGTTTCCATTTGA	*FKS2* mutant construction, hotspot 2 PCR
CgFKS2c4312R	GATTGGATCAGACGTTATACATTG	*FKS2* mutant construction, hotspot 2 PCR/sequence
PDR1u175-F	AACAAGCATAGAGGCGCTGT	*PDR1* amplification
PDR1d110-R	TGAGGTAGTCTAAGTCTCATG	*PDR1* amplification and sequencing

PDR1-c828R	AAGTGACTTAGTGGTGGCAC	*PDR1* sequencing
PDR1-c1703R	GCAACAGCTACATTCAAGACC	*PDR1* sequencing
PDR1-c2600R	CTGCATACTTTGGCACTCT	*PDR1* sequencing
PGK1-F	CAAACGGTGAAAGAAACGAGAA	
PGK1-R	CCGACACAGTCGTTCAAGAAAG	
GCN5-F	GGTGTCTAAACGGCAGAAAG	
GCN5-R	CTCTTCGTCTTCGTCTCTATTG	
EPA13-F	CAACTTTGCCTTTGGCTTTC	
EPA13-R	CTTTCGTTGCGTCGTGACTA	
EPA6-F	GATGGACGGACCCTACTGAA	
EPA6-R	GCCTGTTCGAGGATAATTCG	
BMT5-F	TCCATTGACGATACGTTGGA	
BMT5-R	TCTTTGTCAAACGCTTGTGC	
BMT2-F	ACCGCACAGGAAAATCAATC	
BMT2-R	AAGATGCACCTTCCGTCATC	
HBN1-F	TGATGACAAGACCACCGAAA	
HBN1-R	AAGAGGCTGGGATCTTGGAT	
CAGL0I09108g-F	ACTGGGAACTGGTCTTGGTG	
CAGL0I09108g-R	TTCTTAGGCACACGCTCCTT	
CAGL0L00157g-F	ACTGATGAGCACGGTCACAC	
CAGL0L00157g-R	AGCTTGTGTTGGCTTTTCGT	
FKS1-F	CCTCACTATGCTGAAAGAATT	
FKS1-R	AGGATCTTGATCATCCATACC	
FKS2-F	ATATGTCTGGTTCGTCAACTCC	
FKS2-R	AACGGGACTTTGTGGATCAG	
CDR1-F	TAGCACATCAACTACACGAACGT	
CDR1-R	AGAGTGAACATTAAGGATGCCATG	
CDR2-F	GTGCTTTATGAAGGCTACCAGATT	qRT-PCR verification
CDR2-R	TCTTAGGACAGAAGTAACCCATCT	
CRZ1-F	TTTTGGCGGAGATAATCAGG	
CRZ1-R	AGGCTGCATAAAGGGGCTAT	
STR3-F	GTCACTGGTTGGAGGAAGGA	
STR3-R	TCTTTGATATTGGTATGGAGCC	
CAGL0L06776g-F	CTGCTGATGCAAAAATCCAA	
CAGL0L06776g-R	GCCATAGGGTCCTCTTCTCC	
CAGL0L03828g-F	CGGTGACTTCGTTGACATTG	
CAGL0L03828g-R	CCAACTTACCACCACCTTCG	
CAGL0H09614g-F	CCATCTTCTTCCAAGGCTTC	
CAGL0H09614g-R	TCACCGATTTGAGAGATAGC	
AWP7-F	TGCTCAGAAACCGACAATCA	
AWP7-R	CTGTGATGGCCTCACTAGCA	
ERG11-Fn	ACGGTACCAAGCCATACGAG	
ERG11-Rn	GAACACTGGGGTGGTCAAGT	
PDR1-F	AAAGGGAGTGACAGCGAGAA	
PDR1-R	ATGGCGTCAATGGATGATTT	
CAGL0M03377g-F	TGGGATTCCCGTTTATTCAA	
CAGL0M03377g-R	CGTTGTAGTCACCGCTGAAA	
CAGL0K10626g-F	CAACAAGGTCGGTGGTATTTAC	
CAGL0K10626g-R	ACAGGTTGAAGCTCCTCTGAG	
CAGL0I00484g-F	CTGTAAGTGCGTTGGCTAAG	
CAGL0I00484g-R	CCTCGAATAGAGATGGAGTGA	
SUT2-F	CTAACATCGGTGCCACATTG	
SUT2-R	GCAGACACAAAAGGGGAAGA	

Reference

1. Healey KR, Paderu P, Hou X, Jimenez Ortigosa C, Bagley N, Patel B, Zhao Y, Perlin DS. 2020. Differential Regulation of Echinocandin Targets Fks1 and Fks2 in Candida glabrata by the Post-Transcriptional Regulator Ssd1. J Fungi (Basel) 6.
